# “This is how we should work” - healthcare professionals’ past uncertainty and present confidence in management of fibromyalgia

**DOI:** 10.1080/17482631.2026.2644582

**Published:** 2026-03-19

**Authors:** Anne Marit Mengshoel, Margareta Aurén-Møkleby, Åse Skarbø, Merja Helena Sallinen

**Affiliations:** a Department of Public Health and Interdisciplinary Health Sciences, Institute of Health and Society, Faculty of Medicine, University of Oslo, Oslo, Norway; b Hospital for Rheumatic Diseases, Lillehammer, Norway; c Division of Surgery, Akershus University Hospital, Lørenskog, Norway; d Faculty of Health and Welfare, Satakunta University of Applied Sciences, Pori, Finland

**Keywords:** Fibromyalgia, self-management, stress, personal recovery work, clinical mindline, healthcare professionals’ experience, interdisciplinary work

## Abstract

**Objective:**

Fibromyalgia is a chronic illness that is poorly understood by healthcare professionals (HCPs), and HCPs may express uncertainty due to limited available knowledge. Our objective was to explore what HCPs tell about their present confidence in delivering self-management programs to patients with fibromyalgia.

**Methods:**

A narrative research approach was applied, and interviews were conducted with nine HCPs from various professional backgrounds. The HCPs were experienced in the management of fibromyalgia, and one year earlier, they had revised their self-management program to ascertain person-centredness. The interview data were subjected to a narrative analysis of what and how they storied their clinical experiences of delivering the programs.

**Results:**

The new confidence of the HCPs was grounded in a cohesive, collective platform with an explicit purpose linked to a defined content and an explorative educational approach. This was articulated by a shared clinical mindline emphasizing stress, patients’ experiences of life stress, and facilitating patients to find solutions to repair their disrupted daily lives. The cohesive, collective platform fostered mutual respect and shared responsibility for the wholeness of the program. Further the collective as well as discipline-specific clinical mindlines and skills were bolstered through the team’s engagement and reflective learning on episodes in practice.

**Conclusions:**

The findings indicate that clinical confidence arises from a shared clinical mindline that articulates a theoretical rationale linked to the actions required and how to deliver the program, together with interdisciplinary collaboration and ongoing reflective practice.

## Background

An approach that incorporates multicomponent therapies and involvement of multiple health disciplines is recommended for the management of fibromyalgia (Eich et al., 2012; Jones et al., 2024; Macfarlane et al., 2017), a common chronic widespread musculoskeletal pain condition of unknown aetiology. Fibromyalgia is characterised by a variety of unpredictable, incomprehensible, fluctuating symptoms (Mengshoel, 2022), such as pain, fatigue, cognitive problems, sleep problems, depression, headache, and gastrointestinal problems (Wolfe et al., 2016). The condition is not verified by any clinical laboratory or radiological measures, leading HCPs and others to question the ‘realness' of fibromyalgia (Madden & Sim, 2016; Mengshoel et al., 2018). The recommended primary therapies are non-pharmacological, including patient education, exercise programmes, and cognitive behavioural therapy (Macfarlane et al., 2017). However, these programmes have small to moderate effect sizes (Araya-Quintanilla and Cuyul-Vásquez, 2025), and patients are often dissatisfied with the therapies (Climent-Sanz et al., 2024; Hu et al., 2025). Thus, the patient’s perspective should be taken into account in fibromyalgia management.

Research has shown that HCPs exhibit significant knowledge gaps regarding the diagnosis and management of fibromyalgia (Hayes et al., 2010), and patients perceive that they are inadequately informed about the condition (Wigers et al., 2024). A recent synthesis of qualitative studies shows that HCPs often ignore, dismiss, or belittle patients’ suffering (Bontempo et al., 2025). Another synthesis reports that HCPs hold negative attitudes, prejudices, and stereotypically negative views towards patients, which may contribute to poor communication, delayed diagnosis, and inadequate treatment and rehabilitation (Byrne et al., 2023). For example, in consultations with rheumatologists, patients may feel inadequately understood, receive little compassion, or obtain insufficient information about their diagnosis and management options. Conversely, rheumatologists perceive patients as challenging and time-consuming to treat, which is intensified by the lack of effective treatment (Colmenares-Roa et al., 2016). Both physicians and other HCPs experience uncertainty regarding fibromyalgia and suitable treatment for patients, leading to problematic interactions between HCPs and patients (Briones-Vozmediano et al., 2013). Uncertainty regarding one’s competency can lead to reluctance to treat and care for these patients (Doebl et al., 2020). Therefore, investigating the experiences of professionals seems relevant to learning what is set in motion when HCPs practise the management of fibromyalgia.

Patient education is recommended for patients with fibromyalgia (Macfarlane et al., 2017). In rheumatology, multidisciplinary patient education in self-management was initially developed in the 1980s for individuals with rheumatoid arthritis based on the principle that adopting health behaviour can result in improved health outcomes (Lorig & Holman, 2003). However, subsequent critiques of these self-management programmes underscore concerns that they operate under the assumption that ways of self-managing are universal to patients and that the programme content is predominantly designed and directed by HCPs (Kralik et al., 2004; Paterson et al., 2001). In a project aimed to co-produce a person-centred self-management programme for people with fibromyalgia together with a rehabilitation team at a rheumatism hospital, the researchers encouraged the team to read and discuss qualitative studies of patients’ illness and recovery experiences (Mengshoel & Sallinen, 2019). This shifted their focus from addressing symptom relief and healthy lifestyle to emphasising patients’ life experiences and individuals’ capacities and resources for restoring a disrupted daily life (Mengshoel et al., 2021). One year after participating in the programme, the patients experienced more well days, and they still exercised an agency to repair their daily life (Mengshoel et al., 2025). Initially, the researchers were surprised by the uncertainty the experienced HCPs expressed about their ability to effectively serve these patients. Equally surprising was the confidence they displayed in the interviews one year after the implementation of the newly crafted programme. Thus, our current objective is to examine how HCPs’ experiences may resonate with those of patients by asking what the HCPs tell about their present confidence in the management of fibromyalgia.

## Theoretical framework

Our experience in developing the programme indicated that translating evidence about patients’ illness and recovery experiences into clinical practice was a transformative process in which diverse forms of knowledge were negotiated, constructed, and embodied, reflecting an evolving nature of knowing (Greenhalgh & Wieringa, 2011). Accordingly, it was assumed that professional knowledge is constructed by a mixture of formal theories, practical skills, tacit “know-how” and clinical experiences. Formal theories can be collectively shared across professions, but are often interpreted or practiced differently, and they also include knowledge that is unique to a particular discipline (White et al., 2006). Gabbay and le May (2011) argue that there is a gap between abstract, formal theoretical knowledge and the knowledge needed in use (‘knowledge-in-practice-in-context’) to approach situations in real-world practice. Hence, in clinical practice, theoretical knowledge is exposed to knowledge-in-practice, which together may foster clinical mindlines to effectively navigate clinical situations. Thus, clinical mindlines refer to internalised theories which are shaped by clinicians’ training and their own and others’ experiences (Gabbay & le May, 2011). Currently, the concepts of formal theories, clinical mindlines, and context-specific practical knowledge are analytic sensitising concepts.

Another issue guiding our work is learning through reflections on clinical practice. White et al. (2006) contend that knowing in clinical contexts is not static but evolves through a commitment to reflectivity on what happens in clinical situations. The authors claim that reflective practice comprises reflections on both taken-for-granted general understandings, disciplinary understandings and knowledge-in-practice experiences. However, the existence of professional boundaries may delineate what is considered pertinent for collaborative practitioners. A collaborative reflective process poses a potential threat to established professional identities as it requires individuals to confront and reassess their fundamental beliefs and practices. This encourages a shift away from merely defending traditional disciplinary norms towards actively dismantling preconceived notions and values. This calls for clinical cultures where reflection and collaboration can thrive (White et al., 2006).

In the current study, HCPs’ stories about their practice were explored. A narrative research approach was found appropriate as narrative enquiry focuses on sequence of interrelated accounts within a story that ‘accretes meaning as it goes’ (Squire et al, 2014, p.4), and this meaning shapes how an individual acts (Frank, 2010). Stories include telling about events in clinical real-life settings, which may display how theoretical knowledge and knowing-in-practice are enacted in a clinical context (Mattingly, 1998). Telling a story means to describe actions, choices, and beliefs to illustrate a point to a listener. Consequently, depth and richness of individual accounts are prioritised over maximising sample sizes (Loyal & Amri, 2026; Malterud et al., 2016). Based on a story, a narrative is constructed, capturing temporality through coherence or shifts between past and present, spatiality in context-bound accounts, and sociability because stories are co-constructed (Haydon & van der Riet, 2017). A narrative approach identifies what is the significant points of a story and orders the interconnected accounts to inform the purpose of the study. It is paid attention to the accounts’ structural roles of providing an orientation, key information, evaluation, and it is attended to links between accounts by linguistic phrases such as ‘and then’,’ ‘that’s why’, or ‘therefore’, indicating a narrative causality (Hurwitz, 2000; Riessman, 2008). In health sciences, narrative approaches may bridge theory and practice and reconcile scientific and humanistic perspectives (Butchart & Parsa, 2024).

## Methods

### Design, context and ethics

This study adopted a narrative research approach (Riessman, 2008). To contextualise our data, we utilised our field notes and insights into ongoing discussions at the time the project started. Individual interviews with the HCPs were conducted one year after the implementation of the person-centred self-management programme. HCPs with different professional backgrounds were interviewed individually as we assumed that they had various experiences although working together. The study was undertaken at Lillehammer Hospital for Rheumatic Diseases in Norway. The HCPs worked in the rehabilitation department where diagnose-specific programmes of education in self-management for inpatients and outpatients with rheumatoid arthritis, osteoarthritis, and fibromyalgia have been provided since the 1990s. Participation in interviews was voluntary. Before the interviews began, the researchers informed about the purpose of the study and HCPs’ rights to withdraw from the study at any time, and we promised them anonymity in our further work. The colleagues and others knew who participated in this project, and we therefore omitted person-identifiable information. However, every participant is quoted at least once. The project followed the national legislation and recommendations for medical and health research ethics, and the project was approved by the Norwegian Social Science Data Services (no. 2018/57956/3/EPA).

### Participants

After one year, all HCPs with recent experience of delivering the new programme were asked to volunteer for an interview, and everyone consented (*n *= 9). All but two had been involved in developing the programme. The female participants, aged from their early 30 s to 60 s, had professional backgrounds in medicine (*n* = 2) respectively with specialities in rehabilitation medicine or rheumatology, nutritional counselling (*n* = 1), occupational therapy (*n* = 2), physiotherapy (*n* = 1), psychology (*n* = 1), and social work (*n* = 2). Several allied HCPs had further education in supervisory pedagogy. All participants had considerable experience working with patients suffering from persistent musculoskeletal pain, and most of them had been involved in delivering patient education programmes for patients with fibromyalgia for years, either at the rheumatism hospital or elsewhere.

### Data generation

Individual interviews were conducted by three of the authors. The HCPs were asked to tell about their past and present experiences with the management of fibromyalgia by reflecting on the following: their interpretation of fibromyalgia and their experiences and roles in delivering self-management programmes. The interviews followed a thematic interview guide with the following overarching questions: Could you share your experiences of working with patients with fibromyalgia in the past and today? What do you see as the core features of the programme you deliver today? From your perspective, have you noticed any differences compared with earlier practices? Are there elements of this project transferable to other areas of your clinical work? The interviews lasted approximately 30 to 90 minutes.

### Data analysis

Six interviews were transcribed manually, two interviews were converted from voice to text using the Autotext software programme and controlled for accuracy, and one interview was written down as accurate as possible during and immediately after the interview, as the participant did not volunteer to audio-record. A comprehensive understanding of the data was developed by two authors discussing and reading the transcripts multiple times. Our initial impression was that they contrasted a past uncertainty with a clinical confidence in the present related to the importance of shared understanding, collaboration, and time for reflections. These issues were explored in detail in the further analysis.

Each interview was analysed individually as a story using narrative analysis (Holstein & Gubrium, 2012; Mattingly & Garro, 2000; Riessman, 2008). Our analysis focused on identifying accounts that responded to the questions: What do the individual say about their shared understanding, actions in clinical practice, collaboration, and reflection? The accounts of each interview were organised into sequences of interrelated accounts, in terms of what the information provided (an orientation about the issue, key points explaining the issue, an evaluation or end) and how the language was used as connectors (e.g. “and then,” “therefore,” and “that’s why”) signalling a narrative causality. Individual stories revealed considerable variation in how meaning was constructed. It was searched for refutational meanings, but meanings did not contradict across interviews. Key elements across the stories were used to articulate a collective narrative (Frank, 1995; Holstein & Gubrium, 2012). The analysis proceeded iteratively, moving between the whole texts and their parts and continued during the writing process. Quoted phrases in the text are marked with “...”.

## Results

To some extent, the initial uncertainty in the management of fibromyalgia stemmed from ongoing national and international debates at the time of the project’s start regarding whether fibromyalgia should fall under the domain of rheumatology and be managed in specialist healthcare. This debate was intensified by a governmental report recommending that care for fibromyalgia should be provided in primary healthcare, with referrals to rheumatologists only if the diagnosis is uncertain. However, the rehabilitation team disagreed with this directive, arguing that there were inadequate resources in primary healthcare to provide the multidisciplinary management of fibromyalgia. Additionally, the hospital’s commitment to evidence-based practice prompted doubts about the programme’s scientific validation, following an effect study that found only minor benefits from an earlier version of the programme. Some approaches included in the programme were also questioned as they were considered to abandon a current view within rheumatology favouring ‘active’ approaches to facilitate patients’ independence of healthcare and active lifestyle.

### A cohesive, collective platform underpinning the HCPs’ work

The HCPs describe a significant turning point in the process of programme development, which took place at a meeting just prior to the implementation of the new programme. This meeting is perceived as a marking moment when “things fell into place” through the evolution of a shared purpose of the programme cohering with the themes to be approached. They say that the preceding patient self-management education programme emphasised information about disease pathology, symptom management techniques, and healthy lifestyle, and the approaches were determined by the respective disciplines. However, despite the comprehensive scope of the earlier programme, today, they underscore that it lacked a unified focus and arguments on how their various approaches were important for patients. The HCPs state that their previous fragmented approach has now evolved into a cohesive platform with a joint purpose, explicit themes linked to the purpose, and a shared framework for how to deliver their programme. This transformation is illustrated in the following quote.

*I think there is more coherence within the programme now. We professionals have a more shared platform and understanding of what it should be about and what is important for patients. Before, I had the impression that it was more like: This is mine, and now I'm going to deliver my part, and then, you take your part. And then, we professionals are not quite united which I think is important to create a wholeness out of what we are doing. Now, it's more like the whole team feels a sense of belonging and ownership.*

As expressed in this quote, the HCPs agree that the current programme have a collective platform. This platform is grounded on a shared clinical mindline among the HCPs embodying a shared purpose by addressing life stress supported by biological evidence, which in turn aligns with the themes explored in the various modules thematising evidence of patients’ stressful experiences. Further, the HCPs state that these themes have fostered a collaborative delivery of the programme. For instance, physicians and physiotherapists integrate their education about biological mechanisms in fibromyalgia with practices of relaxation exercises and engagement in physical activity to achieve less tension and more “bodily balance”. Other HCPs describe that they collaborate in aligning their practices, for example, to facilitate patients to discover ways to improve sleep, modify stress in their individual life context, and how to recover energy during the day by implementing small “time-outs”, varying activities, and eating energising food. In this manner, HCPs argue that a focus on life stress promotes synergy across disciplinary perspectives, collaboration, and clarification of their respective contributions. Furthermore, they found that healing daily life brings a more hopeful attitude toward their practice than before:

*I think that the programme also has new elements by bringing in recovery and life stress. That part has been more clearly highlighted now compared to what was in the group before. A little more concrete through our working on it [when developing the programme]. I think that was good. I don't think recovering was a goal before, but more coping with symptoms. Recovering gives hope of getting a better life in a different way, I think. It is not that they necessarily have less symptoms, but that they face the ailments differently. Living better with the ailments does not give symptoms as much focus.*

In many ways, what the HCPs tell they do today is not that different from before; rather, they say that their approaches have become more explicit and clearer by tailoring and adjusting them to modify life stress and enhance an individual’s personal recovery process instead of tailoring it to disease symptoms, coping and a healthy lifestyle. HCPs exemplify this change by, for example, rewording training into the term physical activity. The exercises are remodelled from conditioning exercises for the purpose of pain relief to physical activity to enhance stress management and learn from what the “intelligent body” tries to say about stress. As a reminder to keep their focus on the patient’s learning process on track, the HCPs refer to a “mantra” they have created: “First provide insights, then identify a modifiable problem, then explore the individual’s resources and possibilities, then take action in own life to attain less stress and create a better daily life”. However, learning is not understood to be a linear process. A hand-drawn portrait hanging in the meeting room illustrates an understanding of patients’ learning as a spiralling process, including a potential series of ups and downs that nevertheless move upwards by making sense of the situation, making changes in daily life, to gradually bringing more wellness into life. In this learning process, the role of the HCPs changes from telling patients what to do to facilitating each patient’s journey to reconnect with the alienated body and discover and pave possible ways to navigate daily life.

### A team with mutual respect and shared responsibility for practice

Notably, the HCPs say that they have come to recognise a shared need to deepen their understanding of fibromyalgia and their possible contribution to help patients, as expressed in this quote: “I thought I was the only one who did not know, but nobody knows exactly”. This positions team members as equal in their need for more knowledge. The previously disconnected and discipline-specific organisation of the programme engendered uncertainty among the HCPs, as the disjointed structure had led the HCPs to perceive a compelling obligation to advocate for the significance of their respective disciplines. When telling about the past, a deep-felt personal disciplinary responsibility was articulated:

*I think that before, it was a bit of a competition to want to show that you were good. I think so! A bit ambitious on behalf of own profession. Not that you're worth nothing, but more that you want to be just as good as the others. And some have even said that they felt inadequately competent because the others seemed so professionally skilled and professionally updated and such. I think that's less so now. One understands that there is a shared responsibility to ensure a wholeness of the programme.*

This quote expresses an observation of a shift in professionals’ expectations from having a solitary significance into constituting a part within a collaborating team. HCPs are no longer defensive and competitive, but have become contributors to an incorporated wholeness of practice. Furthermore, they express confidence in their relationships with others by knowing that they are aligning with the same therapeutic route:

*When I present, I know that the others are on the same track. It strengthens interdisciplinarity that we know what we will work on. I have learnt that we can work towards the same goal even though we represent different professions. I think we've all grown in this as a group. We kind of accept that we go into each other's perspectives. Yes, this is just how we work, and I notice that we are agreeing on that. So, this is how we should work!*

This quote illustrates confidence in colleagues and a personal role within a team, and the use of the pronouns “I” and “we” unifies the individual and the group on a collective track. The HCPs said that their way of working with fibromyalgia is transferable to other settings at the hospital. They have a flexible attitude toward professional boundaries. The HCPs no longer defend and protect their own disciplinary knowledge and professional roles that they may sometimes experience in other settings at the hospital. Instead, they work to improve their collective practice and respect and value each other’s knowledge and experiences.

### Cultivating team engagement and reflective learning on knowledge in practice

Challenging episodes, often unexpected new episodes, occur in clinical practice. Instead of expressing uncertainty, HCPs say that they rely on each other’s competency in such situations. They also address such events collectively during short "debrief meetings" at the end of course days. They state that these meetings are crucial to maintaining their commitment, as meetings foster enthusiasm and engagement:

*I feel more so when they started with a new group today, I thought: Yes, I'm going in there in 3 to 4 weeks. Now, I'm curious about: How many people were there? Were they young? So now, I'm looking forward to talking to the others to hear how the day went. Yes, how did you experience it! And when the physiotherapist is involved, did they take part in physical activity? I didn't care about that before.*

The quote exemplifies that the HCPs are attentive to their colleagues’ practice experiences as it matters to the wholeness and to attaching their own contribution to what has come up during earlier course days. This reflects a sense of responsibility for the coherence of wholeness of practice. Debrief meetings also serve as a space for team members to engage collectively in reflective learning. They view practical experience as an important source of knowledge for refining or revising their work. For instance, they described previous common uncertainty shifting to confidence regarding how to address patients’ experiences and respond to their questions:

*My interactions with these patients used to differ from those with other rheumatic patients. When engaging with individuals with fibromyalgia, I felt compelled to proceed with caution. It was like walking on eggshells. They have so many negative experiences with HCPs that I did not want to add to. But now, we ask them to tell and share such experiences, and then we together learn from it.*

*I used to rely on my PowerPoint presentation to limit patient questioning and to retain some semblance of control. After all the scientific papers we have read together and everything we have discussed about patients’ and our own experiences, I have become more confident and assertive in the consultations. Before, it was more like: How can you manage your day? But now, it is more: What can you do to make your day better? Become more assertive, I would say.*

These quotes show a prior reliance on defensive strategies, indicating a deep discomfort with engaging in a dialogue about the negative experiences of patients that the HCPs previously found “draining energy” and difficult to navigate. Currently, the team has developed an assertive approach to patients by acknowledging that they have negative experiences. Today, HCPs describe confidence in engaging in dialogue and turning negative experiences and restrictions into a chance to look for opportunities, which they experience foster patients’ confidence in them:

*Before, I thought the patients were more complex. Now, I see that they’re not more complex than other patients. Thus, I’m very concerned that we do not make it more difficult than it is. It is a relief not to be admonishing what is right and wrong for patients to do. Now, I try to get them to think about their home situation while I talk. How is it like for you? You have a family and children, what are the pressing issues for you here? What can you do about it? Afterwards, they share their thoughts. I have yet to meet anyone who doesn't say anything then.*

This quote reveals that HCPs no longer perceive themselves as the ultimate authorities on what constitutes the best course of action for patients; rather, they express their present expertise in fostering dialogue and helping individuals find personalised solutions reflecting their unique circumstances. This explorative, dialogic approach empowers HCPs, enhances the team’s learning, and bolsters their competencies. Reflecting on their own and each other’s stories about daily practice, reflective learning on practice episodes emerges that moulds knowledge-in-practice-in context and practical skills, and this may change actions in practice. For example, applying anecdotes from own life can establish deeper connections with patients:

*In one of the sessions, I was asked: What is the meaning of life stress? It was difficult to give a good answer to that. I then provided an example from my own life where a tour guide prioritised highlighting picturesque photo opportunities over providing information about the historical buildings. I became upset, and it destroyed my enjoyment of the tour. This was an occasion of life stress to me! My story prompted a lively sharing of experiences among the patients which I found very meaningful and engaging.*

This quote illustrates that the HCPs recognise that sharing and learning from patients’, their own, and colleagues’ experiences and anecdotes is meaningful and bolsters their collective and disciplinary knowledge-in-practice-in context and skills. By valuing one’s own and others’ experiences, fibromyalgia is no longer considered difficult because life stress is a common human experience.

## Discussion

Confidence emerged through a professionally justified collective “platform” with a coherence between the shared purpose, an articulated framework for what to emphasise and how to work, and a collaborative practice marked by mutual respect, shared responsibility, joint engagement, and reflective learning. The narrative plot conveys the conviction: “this is how we should work”.

Frank (1995, p. 22) eloquently stated that ‘the stories we tell about our lives are not only reflections of our experiences but also contribute to what becomes our experience.’ Currently, it is noteworthy how consistent the HCPs’ experiences have become. They have created a cohesive collective clinical mindline in which they agree is crucial for their work. Emphasising patients’ resources and developing patients’ problem-solving skills to manage self-identified problems means a change from an expert-driven approach to a person-centred approach (Berger & Heusser, 2017). Their approach aligns with Frank (1995) quest recovery narrative template describing a person’s efforts to take action to familiarise themselves within an unknown terrain of living a life with illness. Thus, a quest narrative is embedded in the collective clinical mindline and guides how the HCPs approach the patients’ challenges. Life stress and the quest recovery narrative template prompted a more proactive approach among HCPs. Earlier, emphasis was placed on symptom management, coping, and healthy lifestyle behaviours grounded in a disease perspective, where health is understood as the absence or reduction of disease (Blaxter, 2010). Currently, the focus has shifted to individuals’ resources and opportunities to enact changes that improve their everyday lives, reflecting a personal healing orientation (Szawarska, 2025). In contrast to a disease-oriented health concept, here health can be understood as a person’s ability to attain vital life goals (Nordenfelt, 2025). This shift signifies a profound transformation in the perspective of HCPs, although exercise, sleep hygiene, and activity regulation are still included. However, the HCPs no longer assume that patients are victims of a disease who they must teach how to cope, or that they should hold definitive answers about how to provide relief. Instead, they recognise patients as resourceful individuals and experts on what is possible and appropriate to do within their unique life context to heal their life situation. This resonates with the experiences of the patients who, one year after participating in the programme, continue to take agency to repair their disrupted lives and bring more wellness into their daily lives (Mengshoel et al., 2025). This also aligns with the statement that the term self-management should not solely relate to educating patients on technical skills to manage disease, but also to approach the whole person and her/his need to become an agent in one’s own life (Berger & Heusser, 2017).

Van Alboom et al. (2025) argued that the pervasive influence of a biomedical framework within the healthcare system frequently results in patients with fibromyalgia feeling trapped in a network of illness explanations and treatment models. A biomedical framework prioritises expert knowledge and emphasises the supremacy of technological advancements, science, and evidence-based practice. Complementing this perspective, Frank (1995) posits that a biomedical narrative restitution template is the dominant ideal in the culture of medicine, whereas the principal aim of health services is to provide treatment to restore patients to their pre-illness conditions. Thus, the expertise of HCPs and the hallmark of the success of health services are curative effects. When curative treatment is unknown, both HCPs and patients may become narratively lost (Ahlsen et al., 2020). Furthermore, disciplinary boundaries and interpretations may result in multiple understandings of the illness and suggestions for treatment approaches (Liberati et al., 2016). Evidence-based guidelines for the management of fibromyalgia provide neither an explicit purpose and how to practice the management of fibromylgia nor do they consider the ill person’s life experiences (Macfarlane et al., 2017). Currently, the HCPs bridged an understanding of biological “distress” and life stress conceptualising it as a hinder for recovery, and together with a quest recovery narrative template, this collective clinical mind-line facilitated a proactive attitude to fibromyalgia by both HCPs and patients as it concretised the self-management of fibromyalgia by placing the emphasis on concrete daily life events. Patients find it important to be listened to, accepted, and believed (Sallinen et al., 2011), but they also need to determine how to improve their daily life in the face of illness (Eik et al., 2022).

During the development of the new programme, the HCPs indicated that their patients received significant benefits from connecting and sharing experiences with their peers. At the same time, the HCPs expressed that for them the patients’ “illness talk” was emotionally draining. In contrast, today the HCPs consider the patients’ experiences relevant to facilitate patients’ healing work. The HCPs encourage patients to explore and articulate problems in the daily life context and then collaboratively seek possible solutions to adjust or to do changes. In this process, it is an essential component of patients’ learning to voice and hear their own storytelling and to have an audience that actively engages with their story (Charon, 2006). An opinion expressed by HCPs is that experiences of patients with fibromyalgia is familiar to them and do not differ from other patients, and both healthy and ill people share experiences of exposure and response to life stress. With reference to philosopher Kristeva, Wahl and coworkers (2022) argue that human vulnerability unifies people rather than separating healthy from ill persons, as also portrayed currently. Thus, we argue that these HCPs have adopted a humanistic conception of patients as unique individuals (Vogt et al., 2014).

Our findings suggest that interdisciplinary collaboration is more than assembling professionals with diverse expertise. It requires a shared platform underpinned by a coherent, collective clinical mindline that encapsulates the wholeness of practice. In interdisciplinary work, this “platform” may need to be overarching to embrace various disciplines and flexible to be adjustable to learning in practice (Gabbay & le May, 2011). Bliuc and Chidley (2022) contend that a collective narrative united by common goals forms the basis for group formation, encompassing personal and social identities as well as shared moral values. The HCPs deemed stories and anecdotes told by both patients and colleagues as vital resources for them to reflect and improve “knowledge in action” as suggested by both Gabbay and le May (2011) and White et al. (2006). The HCPs exchanged their experiences, thereby reinforcing their collective and disciplinary mindlines and building a reservoir of practical knowledge for action in practice, and thereby, the HCPs bolstered their competencies. The recognition of practice experiences as a source of knowledge meant a shift from passive listening to “illness talk” to actively facilitating, engaging in, and learning from patients’ stories and their experiences to discover possibilities for adjustments or changes. As portrayed in our study, interdisciplinary work has the advantage of entailing access to others’ knowledge and tips to refine both one’s own disciplinary and collective skills. To reach such an advantage, mutual respect and trust among people, as well as a willingness to exchange knowledge with others, and to have a flexible attitude towards disciplinary boundaries seems vital (Gabbay & le May, 2011).

### Methodological considerations

Our interview data provided rich, accounts about HCP’s clinical practice, which we judge to offer sufficient information power (Malterud et al., 2016). The material was generated in dialogue between interviewees and interviewers. A potential limitation is that two interviewers had participated to develop the programme. However, participants largely recounted concrete plausible and sometimes unexpected clinical events, which reduced concerns about confirmatory responses. Despite variation in details, there were no contradictions across stories, allowing construction of a collective narrative about what HCPs view as important for fostering confidence in managing fibromyalgia.

In narrative enquiry, trustworthiness depends on methodological transparency and the plausibility of the narrative (Loh, 2013). Constructed knowledge is imperfect and must be open to appraisal. Thus, we have clarified our theoretical lens, methodological choices, and interpretation. Reflectivity was reinforced by the authors various scientific interests and professional backgrounds in nursing, occupational therapy, and physiotherapy. An author was affiliated with the clinical context and contributed to the process with contextual insight, supporting the plausibility and contextual resonance of the narrative. Transferability concerns whether the narrative helps to understand similar situations in other contexts (Loh, 2013). A cohesive “platform” that explicates why, what, and how practice is conducted is likely to resonate broadly. The identified shared clinical mindline framing self-management around patients’ personal experiences and repairing a disrupted daily life, offers an alternative perspective that may create new management approaches to patients with fibromyalgia.

### Conclusions

To our knowledge, this is the first study to examine an HCP team's confidence in managing fibromyalgia. Clinical confidence appeared to stem from a cohesive collective platform that articulated not only what to do, but also why and how to deliver care. It was also associated with a proactive, collaborative approach rooted in mutual respect and shared responsibility for the wholeness of practice, and the group’s engagement in reflective learning from clinical episodes and experiences bolstering their clinical competencies.

## Data Availability

The interview material is stored in the Services for Sensitive Data at the University of Oslo. Any further underlying data will be made available upon reasonable request.
